# Synergistic Antibacterial Effect of Eugenol and Biogenic Silver Nanoparticles on *Staphylococcus pseudintermedius* Isolated from Canine Keratoconjunctivitis Sicca

**DOI:** 10.3390/molecules30163353

**Published:** 2025-08-12

**Authors:** Weslei Roberto Correia Cabral, Caio Ferreira de Oliveira, Rogerio Giuffrida, Lais Fernanda de Almeida Spoladori, Isabela Madeira de Castro, Guilherme Bartolomeu-Gonçalves, Helena Tiemi Suzukawa, Gabriella Maria Andriani, Gerson Nakazato, Eliandro Reis Tavares, Lucy Megumi Yamauchi, Sueli Fumie Yamada-Ogatta

**Affiliations:** 1 Laboratório de Biologia Molecular de Microrganismos, Departamento de Microbiologia, Universidade Estadual de Londrina, Londrina 86055-900, Brazil; cabral.wesleirc@uel.br (W.R.C.C.); lais.spoladori@gmail.com (L.F.d.A.S.); isabela.mcastro@uel.br (I.M.d.C.); helena.tiemi.suzukawa@uel.br (H.T.S.); mgabriella.andriani@gmail.com (G.M.A.); tavares.eliandro@uel.br (E.R.T.); lionilmy@uel.br (L.M.Y.); 2 Departamento de Medicina, Faculdades de Dracena, Dracena 17910-106, Brazil; caioferreira.biomed@gmail.com; 3 Departamento de Medicina Veterinária, Universidade do Oeste Paulista, Presidente Prudente 19050-920, Brazil; rgiuffrida@unoeste.br; 4 Departamento de Patologia, Análises Clínicas e Toxicológicas, Universidade Estadual de Londrina, Londrina 86038-350, Brazil; guilherme.bartolomeu@uel.br; 5 Departamento de Microbiologia, Universidade Estadual de Londrina, Londrina 86055-900, Brazil; gnakazato@uel.br; 6 Departamento de Medicina, Pontifícia Universidade Católica do Paraná, Londrina 86067-000, Brazil

**Keywords:** antibiofilm, methicillin-resistant *Staphylococcus pseudintermedius*, multidrug resistance, toxicity

## Abstract

Plants are a valuable source of bioactive compounds with therapeutic potential. Antibacterials of natural origin represent a promising and sustainable alternative in the fight against bacterial infections. In addition to being effective against bacterial growth, these natural agents may have lower toxicity and fewer side effects, which reinforces their value in the development of new therapeutic strategies. This study reports on the antibacterial effect of eugenol (EUG) and biogenic silver nanoparticles (bioAgNPs) synthesized using the aqueous extract of *Trichilia catigua* A. Juss. bark, alone or in combination, against planktonic and sessile cells of multidrug-resistant *Staphylococcus pseudintermedius*, one of the main opportunistic pathogens in dogs. EUG and bioAgNPs showed a dose- and time-dependent bactericidal effect on planktonic cells, interfering with cell membrane integrity. The interaction between EUG and bioAgNPs was classified as synergistic or indifferent for planktonic cells. Except for one isolate, the combination exhibited a synergistic effect for biofilms previously formed on abiotic surfaces for 24 h. Both bioactive compounds promoted morphological and ultrastructural changes in *S. pseudintermedius* biofilms. All concentrations of EUG and bioAgNPs in synergistic or indifferent combinations showed reduced toxicity to mammalian cells. These findings suggest that the EUG and bioAgNP combination could be a promising strategy for controlling *S. pseudintermedius* infections.

## 1. Introduction

The spread of multidrug-resistant (MDR) bacteria represents a growing threat to animal and public health [1,2] and significantly affects the antimicrobial therapy within veterinary medicine. The reduced efficacy of conventional antibiotics against resistant microorganisms limits therapeutic options and contributes to increased morbidity, treatment costs and the risk of zoonotic transmission [1,2]. In this scenario, the search for new therapeutic approaches that are effective, safe, and sustainable is urgent [1].

*Staphylococcus pseudintermedius* can colonize the skin, hair, and mucosa of dogs, mainly in the nostrils, mouth, groin, perineum, anus, and reproductive tract [3]. Although less frequently, this bacterial species can also be found as a colonizer or infectious agent of a wide variety of animals, including companion and free-living species of birds and mammals [2]. However, this Gram-positive coagulase-positive coccus is one of the most clinically relevant pathogens in veterinary medicine, being the main etiological agent of dermatological and ophthalmological pathologies in dogs [1,4,5]. In addition, this bacterium has often been associated with infections of the external ear, urinary tract, respiratory tract, reproductive tract, and wounds resulting from surgical procedures [2].

Keratoconjunctivitis sicca (KCS), commonly known as dry eye, is one of the most prevalent corneal disorders in certain dog breeds [6,7]. It is a chronic inflammatory condition of the ocular surface, marked by reduced production of the aqueous portion of the tear film [8]. While the condition has multiple causes, immune-mediated inflammation is the most widely recognized and prevalent underlying factor in affected dogs [8,9].

One of the main challenges in the management of KCS is the occurrence of secondary microbial infections, which often require the inclusion of antimicrobials in therapeutic protocols [9]. However, the growing prevalence of methicillin-resistant *S. pseudintermedius* (MRSP), often associated with resistance to multiple classes of antimicrobials in addition to β-lactams, has hampered the management of these infections and increased the risk of therapeutic failures [1,10]. Moreover, infections caused by MRSP associated with the formation of biofilms represent an even greater challenge, since biofilms provide protection against the action of antimicrobials [11,12] and host defense mechanisms [13], favoring the persistence of the infection. In addition to its clinical relevance in dogs, *S. pseudintermedius* is considered an emerging zoonotic pathogen, with the potential to infect humans in close contact with pets [1,2].

**Figure 1:**
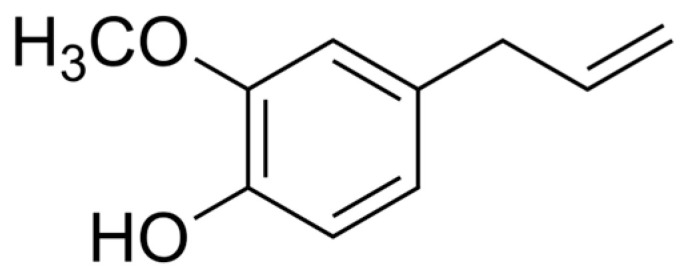
Chemical structure of eugenol (4-allyl-2-methoxyphenol).

Plants have emerged as valuable sources of bioactive compounds with antimicrobial potential [14]. Used for millennia in traditional medicine, plants produce a wide diversity of secondary metabolites—such as alkaloids, flavonoids, terpenoids, and phenolic compounds—many of which exhibit activity against a variety of pathogenic microorganisms, including MDR strains [15,16,17,18,19,20]. In addition, various phytochemicals exhibit different pharmacological effects, combining antimicrobial activity with anti-inflammatory, antioxidant, immunomodulatory, and healing properties, which makes them particularly attractive for the treatment of complex or chronic infections [19]. Among these compounds, eugenol (EUG, 4-allyl-2-methoxyphenol, Figure 1)—a phenolic compound found in several plant species—is widely recognized for its broad spectrum of bioactivity [21]. In the antimicrobial context, EUG has inhibitory activity against planktonic and sessile cells (biofilms) of different bacterial and fungal species [22,23,24].

Plant extracts and their secondary metabolites have also been widely used in the synthesis of metallic nanoparticles [25,26,27,28], a rapidly expanding field in nanotechnology applied to human and animal health. Traditionally, metallic nanoparticle production has been carried out using chemical and physical methods, which, although effective in large-scale production [29], often involve the use of toxic reagents, high temperatures, extreme pressures, and energy-intensive processes. These factors not only increase operating costs but also pose environmental and human health risks. In contrast, biological synthesis, such as that using plant extracts, is emerging as an environmentally friendly and sustainable approach, using natural metabolites such as proteins, enzymes, flavonoids, alkaloids, and phenols as reducing and stabilizing agents [28]. Biogenic silver nanoparticles (bioAgNPs) stand out due to their unique physicochemical properties and multiple pharmacological potential applications, such as antioxidant, anti-inflammatory, antidiabetic, antitumor, and healing [28]. In addition, several studies have shown that bioAgNPs exhibit antimicrobial activity against a wide range of microorganisms, including MDR strains [22,28,30,31,32,33]. Owing to their nanometric size, these particles have a larger contact area and more intense molecular interactions with microbial structures, which contributes to their antimicrobial efficacy even at relatively low concentrations with reduced toxicity to mammalian cells [34].

The antimicrobial activity of EUG and bioAgNPs against *S. pseudintermedius* is still little explored. Furthermore, to our knowledge there have been no studies to date investigating the antimicrobial activity of the combination of these two compounds against this bacterial species. Therefore, the aim of this study was to evaluate the antimicrobial potential of EUG and bioAgNPs, with the nanoparticles obtained by using the aqueous extract from the bark of *Trichilia catigua* A. Juss., alone and in combination against planktonic and sessile cells of *S. pseudintermedius*. The results may contribute to the development of viable alternatives to combat antimicrobial resistance in veterinary medicine.

## 2. Results and Discussion

### 2.1. Antimicrobial Susceptibility Profile of Staphylococcus pseudintermedius

In this study, six *S. pseudintermedius* isolated from dogs with KCS were randomly selected from the bacterial collection of the Clinical Microbiology Laboratory of the Veterinary Hospital of Universidade do Oeste Paulista (UNOESTE). The species identification was confirmed by phenotypic assays [35] and PCR targeting the *nuc* gene [36]. According to the agar disk diffusion assay, four isolates were resistant to oxacillin and harbored the *mecA* gene, as detected by PCR [37], and were therefore classified as MRSP. Two isolates were sensitive to oxacillin and negative for the *mecA* gene, being classified as methicillin-sensitive *S. pseudintermedius* (MSSP). All isolates also displayed resistance to azithromycin, ciprofloxacin, clindamycin, erythromycin, levofloxacin, and penicillin, and were classified as MDR according to the criteria proposed by Magiorakos et al. [38] (Table 1).

There has been a global increase in the frequency of MDR *S. pseudintermedius*, including strains isolated from ophthalmic infections [4,5,26,42,43]. However, data on the antimicrobial susceptibility of *S. pseudintermedius* isolated from KCS are limited in the literature. In a previous study conducted at the same veterinary hospital, *S. pseudintermedius* was the most frequently ocular isolate in dogs with KCS, with three (4.6%) classified as MDR [42]. Supporting this increase in MDR isolates, some studies have reported a high prevalence of multidrug resistance in *S. pseudintermedius* isolated from keratitis in dogs. For instance, Park et al. [4] reported a 52.5% prevalence of MDR isolates, mainly among *Staphylococcus* species, including *S. pseudintermedius* associated with bacterial keratitis in different dog breeds in Korea. Similar results were reported by Casemiro et al. [5] in dogs treated at the specialized ophthalmology services of the veterinary teaching hospital of the State University of São Paulo (UNESP), Brazil. Among all isolates, *Staphylococcus* species accounted for 35.55% and *S. pseudintermedius* was the most prevalent. In addition, approximately 50% of all bacterial isolates were classified as MDR. These findings highlight the urgent need for new strategies to control infections caused by *S. pseudintermedius* in dogs.

### 2.2. Eugenol and Biogenic Silver Nanoparticles Exhibit a Dose-Dependent Bactericidal Activity Against Planktonic Cells of MDR Staphylococcus pseudintermedius

The antibacterial activity of EUG and bioAgNPs against planktonic cells of all *S. pseudintermedius* isolates was initially assessed by the broth microdilution assay [39]. For EUG, the minimum inhibitory concentrations (MICs) were 0.67 mg/mL (two isolates) and 1.33 mg/mL (four isolates). Similarly, bioAgNPs showed an MIC of 3.45 µg/mL against all isolates (Table 1).

**Figure 2:**
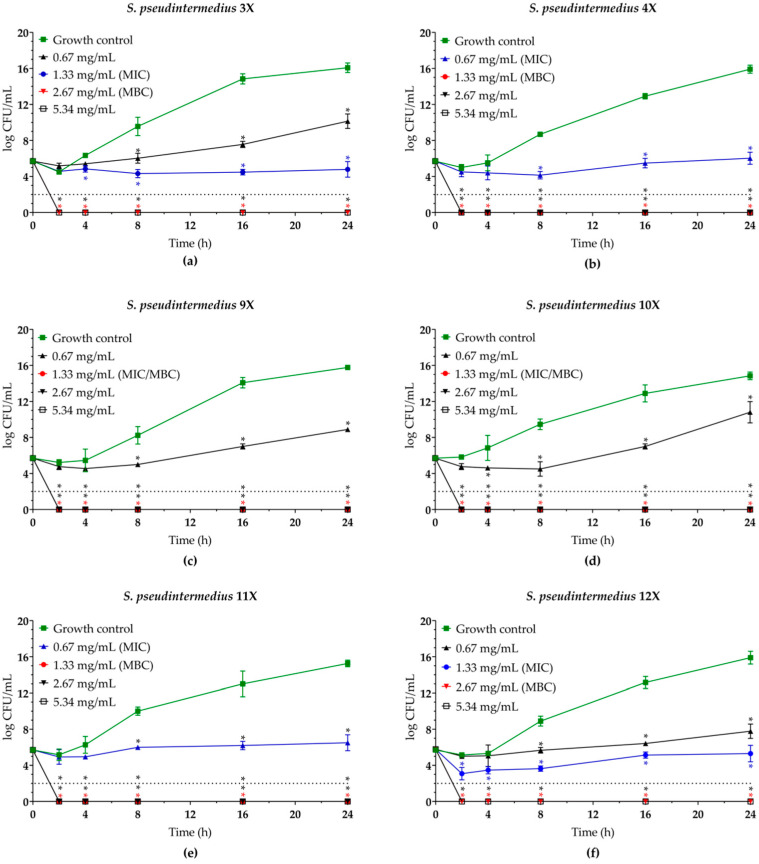
Antibacterial activity of eugenol (EUG) against multidrug-resistant *Staphylococcus pseudintermedius*. Time–kill kinetics of (**a**) MRSP SIG 3X, (**b**) MRSP SIG 4X, (**c**) MRSP 9X, (**d**) MRSP 10X, (**e**) MSSP SIG 11X, and (**f**) MSSP SIG 12X incubated with different concentrations of EUG. The log10 CFU/mL values represent the mean ± the standard deviation from three independent experiments. The dotted lines indicate a 99.9% (3 log10) reduction in CFU/mL counts. *(*p* < 0.05) compared to the untreated control cells.

**Figure 3:**
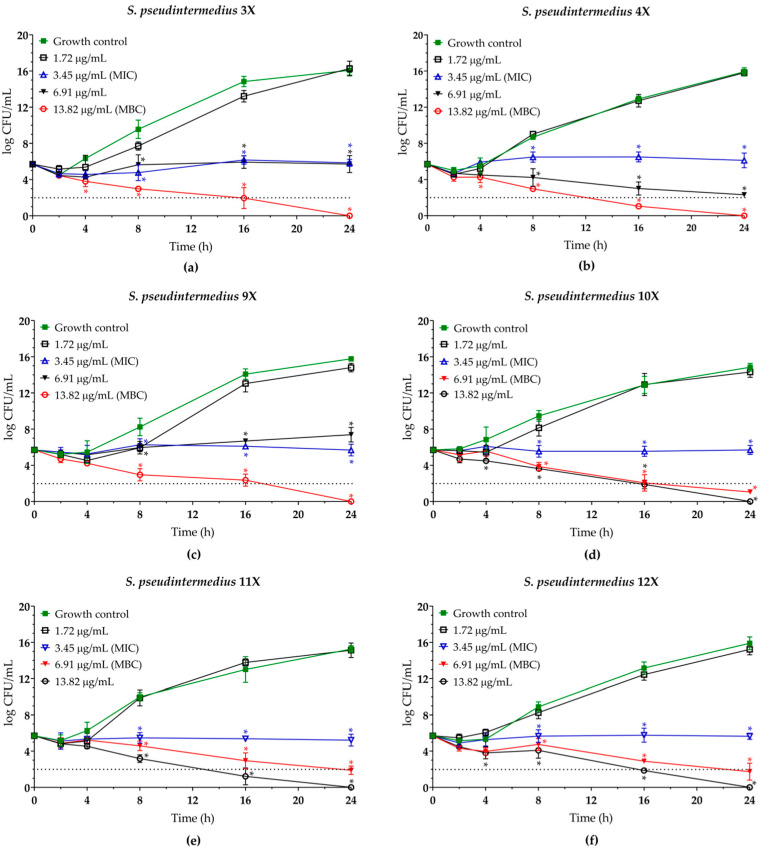
Antibacterial activity of biogenic silver nanoparticles (bioAgNPs) against multidrug-resistant *Staphylococcus pseudintermedius*. Time–kill kinetics of (**a**) MRSP SIG 3X, (**b**) MRSP SIG 4X, (**c**) MRSP 9X, (**d**) MRSP 10X, (**e**) MSSP SIG 11X, and (**f**) MSSP SIG 12X incubated with different concentrations of bioAgNPs. The log10 CFU/mL values represent the mean ± the standard deviation from three independent experiments. The dotted lines indicate a 99.9% (3 log10) reduction in CFU/mL counts. *(*p* < 0.05) compared to the untreated control cells.

To determine the minimum bactericidal concentration (MBC) of both compounds and assess the kinetics of bacterial killing, planktonic cells of *S. pseudintermedius* were incubated in the presence of EUG (0.67 to 5.34 mg/mL) and bioAgNPs (1.72 to 13.82 µg/mL) at 37 °C for up to 24 h. Overall, a dose-dependent inhibitory activity of EUG (Figure 2) and bioAgNPs (Figure 3) was observed. Both compounds, at their MIC values, inhibited the growth of all bacterial isolates over time compared to untreated control cells. The MBC values for EUG were 1.33 mg/mL (four isolates) and 2.67 mg/mL (two isolates). These MBC values led to a 100% reduction in colony-forming unit (CFU) counts after 2 h of incubation for all isolates, indicating rapid bactericidal action. In contrast, the MBC values for bioAgNPs were 6.91 µg/mL (three isolates) and 13.82 µg/mL (three isolates), promoting total elimination of bacteria only after 24 h of exposure, exhibiting slower but effective bactericidal activity. The MBC/MIC ratios were 1.0 and 2.0 for EUG, and 2.0 and 4.0 for bioAgNPs, supporting the bactericidal effect [41] (Table 1).

Despite the extensive documentation on the antimicrobial activity of EUG against various microorganisms [23], there are still few studies investigating its inhibitory effect on *S. pseudintermedius*, as well as on the medicinal plants naturally rich in this phytochemical. The crude ethanolic extract of *Piper betle* L. leaves, whose main phytochemicals are EUG and hydroxychavicol, showed a bactericidal effect against MSSP and MRSP isolated from canine pyoderma. The average MIC and MBC values were 216.27 and 295.63 µg/mL for the MSSP isolates and 212.37 and 312.50 µg/mL for the MRSP isolates, respectively [17]. These findings are corroborated by the study conducted by Jantorn et al. [16], which also reported the bactericidal activity of the ethanolic extract of the same plant species against MSSP and MRSP isolated from dogs, describing MIC and MBC values of 250 µg/mL. In contrast, Silva et al. [44] reported MIC and MBC values of 2000 µg/mL for EUG against *S. pseudintermedius* isolated from canine otitis, which were similar to those observed in the present study. The discrepancy between these values and those observed for the crude ethanolic extract of *P. betle* leaves indicates possible synergistic interactions between EUG and other compounds within the crude extract.

AgNPs have also displayed antimicrobial activity against a variety of microorganisms [28,29,30,31,32,33]. However, there is still a scarcity of studies focused specifically on *S. pseudintermedius*. The AgNPs, obtained by both chemical [43,45,46] and biological [26,27] synthesis, showed inhibitory activity against *S. pseudintermedius*, although the MIC and MBC values varied between the studies. The bioAgNPs used in our study were synthesized using an aqueous extract of the bark of *T. catigua* A. Juss. (*Meliaceae* family). According to the technical report [25], these nanoparticles present a spherical morphology, with dimensions ranging from 60 to 100 nm and zeta potential of −23.3 mV, as determined by dynamic light scattering analysis. The shape and dispersion of the bioAgNPs were confirmed by transmission electron microscopy. X-ray diffraction characterization revealed a single-phase crystal pattern, evidenced by sharp, well-defined peaks at diffraction angles corresponding to the orthorhombic pattern ICSD 01-074-2076. These results are shown in Supplementary Figure S1.

Previous studies using chemically synthesized AgNPs against *S. pseudintermedius* reported particle sizes ranging from 4.69 ± 1.56 nm to 15.00 ± 2.70 nm. These nanoparticles exhibited MIC values between 2.00 and 16.00 µg/mL, and MBC values ranging from 2.00 to 128.00 µg/mL [43,45]. Regarding bioAgNPs, Meroni et al. [26] compared the antimicrobial activity of nanoparticles synthesized from the aqueous extract of *Curcuma longa* (Cl-bioAgNPs) and from the cell-free supernatant of *Escherichia coli* (Ec-bioAgNPs). In both synthesis methods, the bioAgNPs showed a spherical morphology, with average sizes of 11.1 ± 2.75 nm for Cl-bioAgNPs and 27.28 ± 2.68 nm for Ec-bioAgNPs. The MIC values observed against *S. pseudintermedius* were 143.7 ± 0 µg/mL for Cl-bioAgNPs and 3.75 ± 3.65 µg/mL for Ec-bioAgNPs. Additionally, bioAgNPs obtained from the methanolic extract of *Syzygium cumini* leaves also showed a spherical morphology, but with a significantly larger average size (274.6 nm), and MIC and MBC values of 250 and 1000 µg/mL, respectively [27]. These results highlight how the synthesis method, reducing agent, and nanoparticle size affect antimicrobial efficacy against *S. pseudintermedius*.

Although the mode of action of EUG and bioAgNPs on *S. pseudintermedius* was not explored in depth in this study, their action on cell membrane integrity was assessed by fluorescence microscopy, using differential staining with SYTO 9™ and propidium iodide probes. The untreated planktonic cells exhibited green fluorescence, characteristic of cells with intact membranes and metabolically active status. In contrast, cells exposed to the MBC of EUG and bioAgNPs showed red fluorescence, indicating loss of membrane integrity and impaired metabolic activity (Supplementary Figure S2).

The interaction mechanisms and mode of action of EUG and AgNPs are complex and multifactorial. Due to its lipophilic nature, EUG disrupts bacterial membranes by reducing the content of unsaturated fatty acids [47], destabilizing the lipid bilayer, and leading to cytoplasmic leakage and loss of homeostasis [15,22].

Direct contact between AgNPs and target cells is essential to increase intracellular bioavailability and potentiate the toxicity of silver ions. In fact, AgNPs accumulate on the cell wall, forming pores and disrupting the cell membrane. This compromises cell integrity, leading to cell death. However, evidence from the literature indicates that the antibacterial activity of AgNPs is influenced by various physicochemical characteristics, such as size, surface charge and concentration [48]. In addition, Gram-positive bacteria tend to be more resistant to AgNPs than Gram-negative bacteria [49]. A recent study integrated data from metalloproteomics, systematic biochemical analysis, and structural biology, identifying 38 proteins with affinity for silver ions in *Staphylococcus aureus*. Most of these proteins are involved in the oxidative stress response, followed by processes related to energy metabolism. Therefore, silver ions can act primarily on the glycolytic pathway, inhibiting essential enzymes, as well as promoting the accumulation of reactive oxygen species (ROS) through dysfunction of the redox homeostasis system in the final stages of exposure to AgNPs [50]. At high concentrations, ROS can cause DNA and cell membrane damage and induce lipid peroxidation and protein oxidation, culminating in cell death [48].

### 2.3. Eugenol Displays Synergistic Antibacterial Interaction with Biogenic Silver Nanoparticles Against Planktonic Cells of MDR Staphylococcus pseudintermedius

EUG is widely used in pharmaceutical, cosmetic, food, and hygiene products due to its safety at low concentrations. Actually, EUG is recognized as a GRAS (Generally Recognized as Safe) substance for use as a flavoring agent in food [51]. According to Joint FAO/WHO Expert Committee on Food Additives [52], the maximum permitted daily intake is 2.5 mg/kg of body weight. Despite its proven antimicrobial action, the isolated use of EUG may require relatively high concentrations (≥1600 µg/mL) to achieve efficacy against resistant bacterial strains [23], which may limit its safe application in certain contexts. Therefore, in addition to the search for new molecules with antimicrobial activity, the combined use of drugs has become an effective strategy to optimize therapeutic outcomes and delay the emergence of resistance [53]. The combination of two antimicrobial agents may produce a synergistic effect, thereby enhancing therapeutic efficacy and permitting the use of lower doses of each compound. This strategy can minimize the risk of treatment-associated toxicity [53]. In fact, the combined therapy has consolidated applications in human and veterinary medicine. For example, the combination of the β-lactam antibiotic amoxicillin with the β-lactamase inhibitor clavulanic acid is commonly recommended for the treatment of canine pyoderma [54].

**Figure 4:**
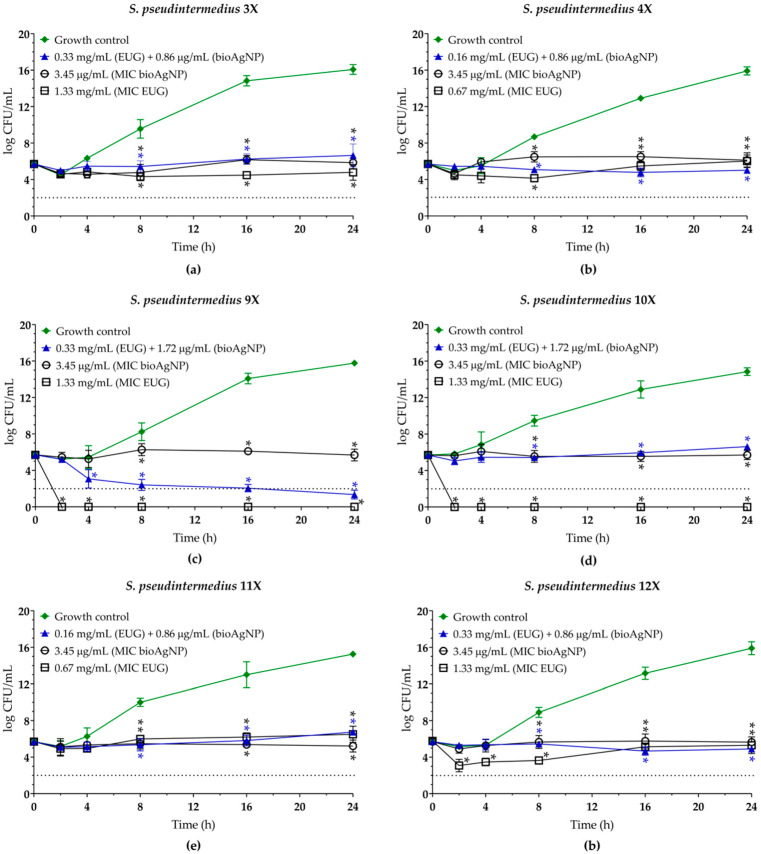
Antibacterial activity of eugenol (EUG) combined with biogenic silver nanoparticles (bioAgNPs) against multidrug-resistant *Staphylococcus pseudintermedius*. Time–kill kinetics of (**a**) MRSP SIG 3X, (**b**) MRSP SIG 4X, (**c**) MRSP 9X, (**d**) MRSP 10X, (**e**) MSSP SIG 11X, and (**f**) MSSP SIG 12X incubated with the minimum inhibitory concentration (MIC) of each compound alone and at the interaction (synergistic or indifferent) concentrations. The log10 CFU/mL values represent the mean ± the standard deviation from three independent experiments. The dotted lines indicate a 99.9% (3 log10) reduction in CFU/mL counts. *(*p* < 0.05) compared to the untreated control cells.

In this study, the antibacterial effect of the EUG/bioAgNPs combination against MDR *S. pseudintermedius* was evaluated using the checkerboard assay [30], and the results are presented in Table 2. The combination of both compounds resulted in a four-fold reduction in the MIC of EUG, and a two- to four-fold reduction for bioAgNPs. The calculated fractional inhibitory concentration indexes (FICI) values were 0.49, 0.50, and 0.79 across the isolates tested, indicating an indifferent antibacterial effect for two isolates and a synergistic effect for the other four *S. pseudintermedius* [55]. Simultaneous administration of the two compounds at synergistic or indifferent concentrations inhibited bacterial growth over time, showing a sustained bacteriostatic effect similar to the growth kinetics observed with each compound alone at MIC (Figure 4).

Even though the classification of antibacterial agents as bacteriostatic or bactericidal provides useful information about their in vitro activity, this distinction alone is insufficient to predict their clinical efficacy. For instance, specific bacteriostatic antibacterials, such as linezolid, remain valuable options, particularly in cases of Gram-positive bacteremia [56]. For a more robust assessment of the therapeutic response in vivo, further studies are necessary to integrate these data with pharmacokinetic and pharmacodynamic parameters. These parameters, respectively, reflect the concentrations of the compounds in the organism and their interactions with the infectious agent over time [57].

Although the mechanism underlying the synergistic antibacterial effect of the combination of EUG and bioAgNPs was not the primary focus of this study, some findings may contribute to understanding this interaction: (i) The antimicrobial activity of EUG is widely attributed to the presence of a free hydroxyl group in its chemical structure. This functional group increases the polarity of the molecule, enhancing its interaction with the bacterial plasma membrane bilayer and consequently increasing membrane permeability [22,23]. (ii) The hydroxyl group also contributes to the intracellular generation of reactive oxygen species (ROS), which can lead to oxidative damage within bacterial cells [58]. (iii) BioAgNPs are similarly known to compromise the integrity of bacterial plasma membranes [28,32,33], although their effectiveness may be reduced against Gram-positive bacteria, which typically exhibit greater resistance to this type of nanoparticle [49]. (iv) BioAgNPs have also been shown to promote ROS generation, further contributing to bacterial cell stress and damage through oxidative mechanisms [50,59]. Based on these observations, we hypothesize that EUG enhances membrane permeability to bioAgNPs, thereby increasing the intracellular availability of both agents. This interaction may amplify ROS production, resulting in cumulative damage to bacterial DNA, proteins, and lipids. Nonetheless, further studies are required to elucidate the molecular mechanisms governing this synergistic effect.

### 2.4. Eugenol, Alone or Combined with Biogenic Silver Nanoparticles, Inhibits the 24 h Biofilms of MDR Staphylococcus pseudintermedius on Abiotic Surfaces

To evaluate the antibiofilm activity of the compounds alone or in combination, the ability of all *S. pseudintermedius* to form biofilms on the polystyrene surface was initially analyzed. The biomass of the 24 h biofilms was evaluated after staining with crystal violet. The mean optical density at 570 nm (OD_570nm_) ± standard deviation was 0.296 ± 0.108, ranging from 0.103 ± 0.029 to 0.570 ± 0.036. According to the criteria established by Stepanovic et al. [60], all isolates were classified as strong biofilm formers under the conditions analyzed (Supplementary Figure S3).

**Figure 5:**
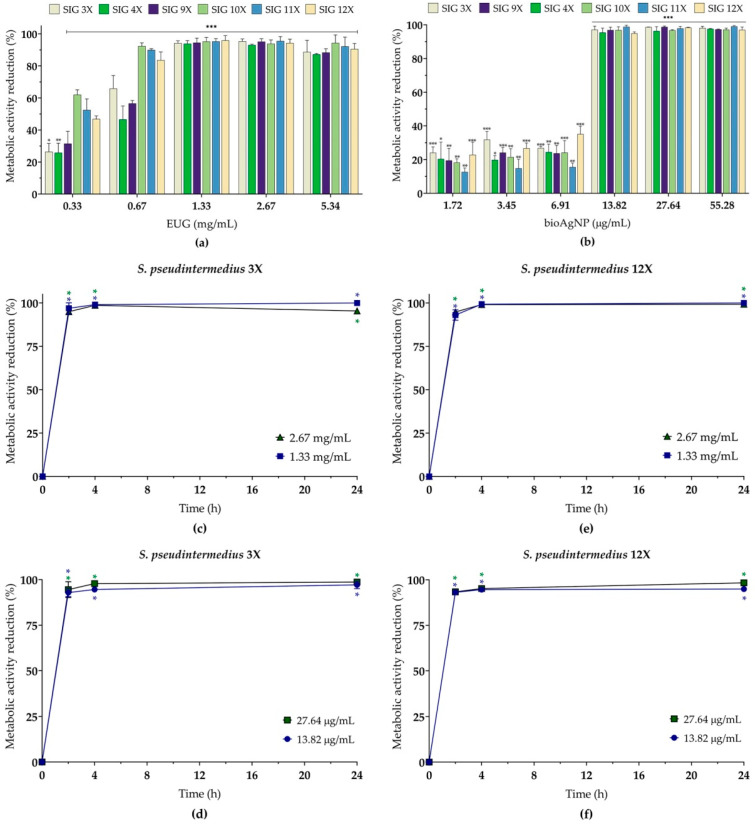
Effect of eugenol (EUG) and biogenic silver nanoparticles (bioAgNPs) on the metabolic activity of sessile (biofilms) cells of multidrug-resistant *Staphylococcus pseudintermedius*, as assessed by the MTT reduction assay. The inhibitory activity of EUG (**a**) and bioAgNPs (**b**) on 24 h biofilms formed on polystyrene surfaces. Time–kill kinetics for (**c**,**d**) MRSP SIG 3X and (**e**,**f**) MSSP SIG 12X isolates incubated with EUG (**c**,**e**) and bioAgNPs (**d**,**f**). Values represent the mean ± the standard deviation derived from three independent experiments. *(*p* < 0.05), **(*p* < 0.01), ***(*p* < 0.001) compared to the untreated control cells.

Subsequently, the inhibitory activity of EUG (0.33 to 5.34 mg/mL) and bioAgNPs (1.72 to 55.28 µg/mL) was evaluated individually on pre-formed (24 h) biofilms (sessile cells), with the results presented in Figure 5a (EUG) and Figure 5b (bioAgNPs). All tested concentrations of both compounds significantly reduced the metabolic activity of sessile cells, with inhibition percentages ranging from 13% to 99%, depending on the isolate and concentration tested. The MIC of EUG capable of inhibiting 90% of the metabolic activity of sessile cells (SMIC_90_) were 0.67 mg/mL (two isolates) and 1.33 mg/mL (four isolates), which were similar to those MICs for planktonic cells. As for bioAgNPs, the SMIC_90_ was 13.82 µg/mL for all isolates (Table 3).

To evaluate the killing kinetics of sessile cells in the presence of both compounds alone, 24 h biofilms of the *S. pseudintermedius* SIG 3X (MRSP, Figure 5c,d) and SIG 12X (MSSP, Figure 5e,f) isolates were treated with EUG (1.33 and 2.67 mg/mL) and bioAgNPs (13.82 and 27.64 µg/mL). All concentrations of EUG and bioAgNPs led to a reduction of more than 90% in the metabolic activity of the biofilms in both isolates after 2 h of incubation.

**Figure 6:**
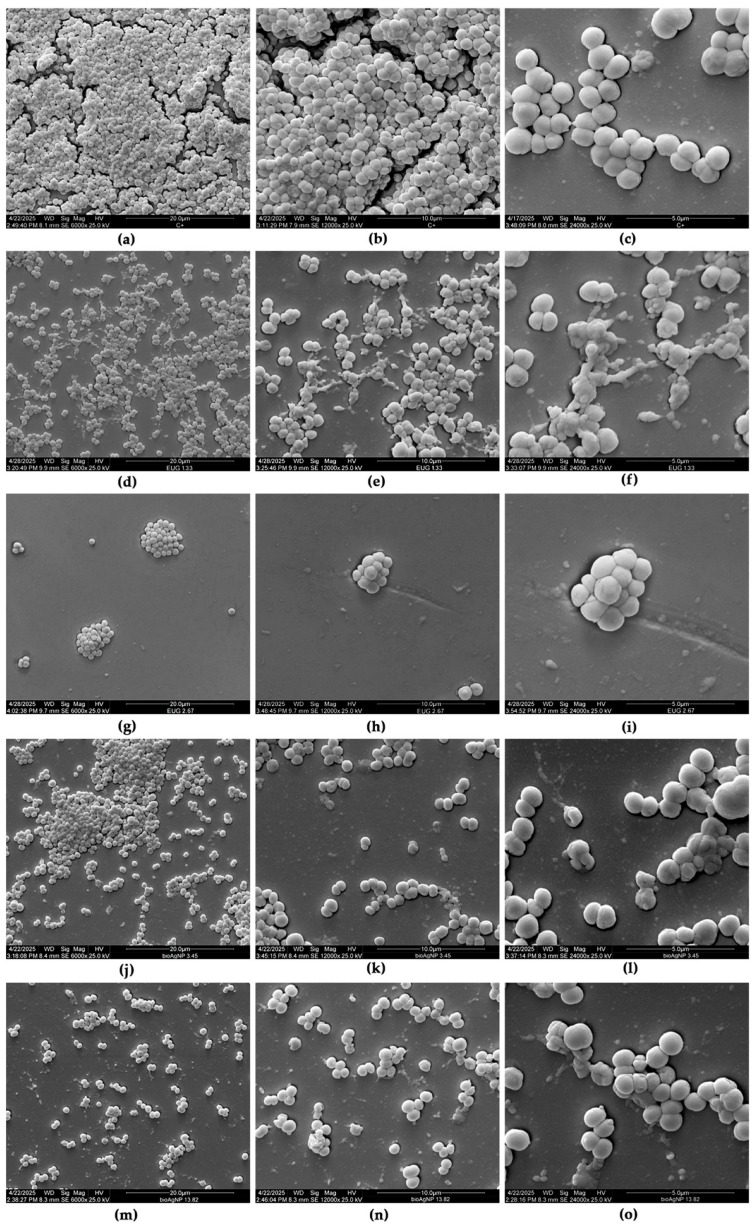
Effect of eugenol (EUG) and biogenic silver nanoparticles (bioAgNPs) on the morphology and ultrastructure of 24 h biofilms of multidrug-resistant *Staphylococcus pseudintermedius* SIG 3X formed on polystyrene surfaces. (**a**–**c**) Untreated biofilms (control). Biofilms incubated with (**d**–**f**) 1.33 mg/mL and (**g**–**i**) 2.67 mg/mL EUG, and (**j**–**l**) 3.45 µg/mL and (**m**–**o**) 13.82 µg/mL bioAgNPs. (**a**,**d**,**g**,**j**,**m**) 6000× magnification; (**b**,**e**,**h**,**k**,**n**) 12,000× magnification; (**c**,**f**,**i**,**l**,**o**) 24,000× magnification.

The effect of the compounds on the morphology and ultrastructure of *S. pseudintermedius* SIG 3X biofilms was analyzed by scanning electron microscopy (SEM). The untreated control biofilm displayed a typical three-dimensional architecture after 24 h of formation on the glass surface characterized by a dense structure, composed of spherical cells organized in multiple layers (Figure 6a–c). Treatment with 1.33 mg/mL (Figure 6d–f) and 2.67 mg/mL (Figure 6g–i) of EUG led to a significant dose-dependent reduction in the biomass of sessile cells. In addition to the reduction in cell density, morphological changes were observed, such as cellular disaggregation, wilted cells, and the presence of amorphous material, suggesting cellular lysis (Figure 6d–i). At the highest concentration tested (2.67 mg/mL), EUG promoted an almost complete reduction in biofilm biomass. Similarly, treatment with bioAgNPs also caused a substantial reduction in biofilm biomass (Figure 6j–o), along with the observation of cells with a distorted and collapsed morphology (Figure 6l,o).

To date, there are no reports in the literature that directly evaluate the inhibitory activity of EUG on *S. pseudintermedius* biofilms. However, the study conducted by Jantorn et al. [16] demonstrated that the ethanolic extract of *P. betle* L. leaves—of which eugenol is one of the main constituents—showed significant antibiofilm activity against this species. The extract was able to inhibit biofilm formation and promote the eradication of biofilms previously established on abiotic surfaces. In addition, molecular docking analyses indicated that eugenol has a high binding affinity for the enzyme *N*-acetylamino glucosamine transferase, an enzyme encoded by the *icaA* gene. This enzyme is involved in the synthesis of poly-*N*-acetylglucosamine (PNAG), the main component of the extracellular matrix of biofilms formed by *Staphylococcus* spp. [61]. Similar results were previously described by Yadav et al. [62], who reported that EUG inhibited biofilm formation and eradicated 24 h biofilms of methicillin-sensitive and methicillin-resistant *S. aureus* strains, at concentrations corresponding to the MIC (0.04%) and 2 × MIC (0.08%) for planktonic cells. Consistent with our findings, the biofilm cells were eliminated in the early stages of exposure (after 3 to 6 h) to EUG. Moreover, previous studies have shown that EUG [18] and bioAgNPs [31] inhibit the expression of key genes of quorum sensing systems in bacteria, which are fundamental to various cellular processes, including biofilm formation.

The antibiofilm activity of bioAgNPs has been widely reported against biofilms formed by different bacterial species [22,28,30,31,32,33,63]. However, to date, only the study by Seo et al. [46] has investigated the inhibitory activity of chemically synthesized AgNPs on *S. pseudintermedius* biofilms. In that study, the authors evaluated the effect of AgNPs on 10 clinical isolates obtained from dogs with otitis externa. The results indicated that the spherical nanoparticles, with an average size of 10 nm, significantly inhibited biofilm formation in a dose-dependent manner, at concentrations of 10 and 20 µg/mL. In addition, SEM images confirmed the reduction in biofilm biomass formed on the glass surface, as well as showing the disruption of the extracellular matrix after treatment with AgNPs.

In the present study, the combined effect of EUG and bioAgNPs was also evaluated on pre-formed (24 h) biofilms of *S. pseudintermedius*. The concurrent addition of EUG and bioAgNPs resulted in a synergistic antibiofilm interaction for most isolates, with FICI values of 0.49 or 0.50. With the exception of SIG 10X, for which the SMIC_90_ of EUG was reduced two-fold, all combinations resulted in a four-fold reduction in the SMIC_90_ of both compounds. This is an important finding of this study, as there are no records in the literature describing the synergistic antibiofilm action between EUG and bioAgNPs. The identification of this synergistic interaction offers new possibilities for the development of more effective antimicrobial strategies against infections associated with biofilms.

### 2.5. Eugenol and Biogenic Silver Nanoparticles Exhibit Reduced Toxicity Toward LLC-MK2 Cells at Concentrations Corresponding to Synergistic or Indifferent Combinations Against Planktonic and Sessile Cells of MDR Staphylococcus pseudintermedius

Evaluating the toxicity of a drug candidate is a fundamental stage in the development of new pharmaceutical formulations. Evidence from the literature, obtained through in silico studies (ADMETox—Absorption, Distribution, Metabolism, Excretion, and Toxicity), indicates that EUG has good oral bioavailability, the ability to cross the blood–brain barrier, and properties compatible with the drug-likeness criteria [64]. In addition, EUG shows good skin permeation and can act as a permeation enhancer [65]. As mentioned before, EUG is recognized as a GRAS substance for use as a flavoring agent in food, as long as the established conditions of use are respected [51]. However, at high doses, it can have adverse effects, including liver toxicity, anesthetic action, and the potential for skin sensitization [65].

Furthermore, one of the main limitations associated with the use of silver nanoparticles (AgNPs) in human and animal health applications is their toxicity. In fact, AgNPs have the ability to translocate and accumulate in various tissues of the body, such as the lungs, heart, liver, kidneys, and nervous tissue, where they can interfere with cell metabolism and induce toxic effects [66]. In view of these, the toxicity of bioAgNPs requires careful evaluation in formulations that combine such nanomaterials with bioactive compounds such as EUG.

**Figure 7:**
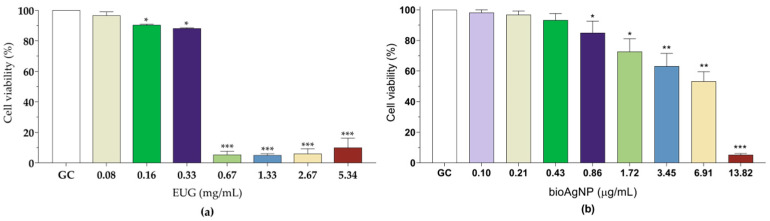
Effect of eugenol (EUG) and biogenic silver nanoparticles (bioAgNPs) on LLC-MK2 cell viability, as assessed by MTT reduction assay. LLC-MK2 cells were treated for 24 h with different concentrations of (**a**) eugenol and (**b**) bioAgNPs. Values represent the mean ± the standard deviation from three independent experiments. *(*p* < 0.05), **(*p* < 0.01), ***(*p* < 0.001) compared to the untreated control cells.

In the present study, LLC-MK2 cells (*Macaca mulatta* kidney epithelial cells) were used as an initial model to evaluate the toxicity of EUG and bioAgNPs to mammalian cells. Therefore, the effect of the compounds on the viability of these cells was assessed using the MTT reduction assay. After 24 h of incubation, it was observed that only around 5.0% of the cells remained metabolically active in the presence of concentrations corresponding to the MIC, MBC, and SMIC_90_ of EUG (Figure 7a). For bioAgNPs, approximately 63.2%, 53.3%, and 5.3% of viable cells were observed at concentrations 3.45 (MIC), 6.91 (MBC), and 13.82 (MBC/SMIC_90_) µg/mL, respectively (Figure 7b). On the other hand, most of the cells remained metabolically active after 24 h of treatment with 0.16 mg/mL (90.4%) or 0.33 mg/mL (88.2%) of EUG (Figure 7a), which correspond to the concentrations in the synergistic or indifferent combinations for planktonic and sessile cells. Similarly, at concentrations of 0.86 µg/mL and 1.72 µg/mL of bioAgNPs, around 85.0% and 72.7% of the cells were viable, respectively (Figure 7b).

A limitation of the present study is that the toxic concentrations of the compounds were not evaluated in canine lineage cells. In this context, Zhang et al. [67] reported a cytotoxic concentration for 50% of the cells (CC_50_) of EUG of 0.165 ± 0.112 mg/mL in primary canine hepatocytes, a value close to the inhibitory concentrations observed in this study, highlighting the importance of more specific investigations in canine cell models. Despite this, evidence indicates that EUG may exert protective effects against toxicity induced by AgNPs. The intraperitoneal administration of these nanoparticles at a dose of 2 mg/kg for 30 days in *Rattus norvegicus* caused intense oxidative stress in kidney and liver tissues, resulting in significant histopathological changes in these organs. However, concomitant oral administration of EUG at a dose of 100 mg/kg body weight was effective in mitigating most of the toxic effects caused by AgNPs. Furthermore, EUG, when administered alone at the same concentration, did not induce changes in oxidative stress markers, biochemical parameters of kidney and liver function, as well as the morphological and immunohistochemical features of both organs [68,69]. Conversely, a randomized clinical study involving 207 dogs naturally infected with the distemper virus evaluated the efficacy and safety of treatment with 3% AgNPs, administered orally and nasally. No significant adverse reactions were observed in the groups treated with AgNPs. In addition, the use of the nanoparticles resulted in a significant increase in the survival rate, both in dogs with neurological manifestations and in those with non-neurological symptoms of the disease, compared to the animals that received only clinical supportive treatment [70].

Further studies are needed to confirm the safety and therapeutic potential of combining EUG with bioAgNPs in the treatment of infections caused by MDR *S. pseudintermedius* in dogs.

## 3. Materials and Methods

### 3.1. Chemicals and Culture Media

Eugenol (EUG, 4-Allyl-2-methoxyphenol, ≥98%) purity was acquired from Ferquima, Vargem Grande Paulista, Brazil); 3-(4,5-dimethylthiazol-2-yl)-2,5-diphenyltetrazolium bromide (MTT), dimethyl sulfoxide (DMSO), Dulbecco’s Modified Eagle medium (DMEM), glutaraldehyde, hexamethyldisilazane, L-glutamine, menadione, sodium cacodylate, penicillin, streptomycin, and tylosin were acquired from Sigma-Aldrich/Merck (São Paulo, Brazil). LIVE/DEAD^®^ BacLight™ kit, Oxoid™ disks of azithromycin (AZM), ciprofloxacin (CIP), clindamycin (DA), chloramphenicol (CHL), erythromycin (ERY), gentamicin (CN), levofloxacin (LEV), oxacillin (OX), penicillin (P), and tetracycline (TE) were acquired from Thermo Fisher Scientific (São Paulo, Brazil). Fetal bovine serum was acquired from Nova Biotecnologia (São Paulo, Brazil). Cation-Adjusted Muller Hinton Broth (CaMHB) and BD Mannitol Salt Agar (MSA) were acquired from BD BBL™ (São Paulo, Brazil). Mueller Hinton agar (MHA), Tryptone Soya Broth (TSB), and Tryptone Soya Agar (TSA) were acquired from HiMedia (Mumbai, India).

Biogenic silver nanoparticles (bioAgNP) were obtained after AgNO_3_ reduction by the aqueous extract of *T. catigua* A. Juss. bark, a process that was patented (BR 102021016375-5) [25] and were acquired from GRAL Bioativos^®^ LDTA (Nano VerdeAg^®^, Londrina, Brazil). Briefly, air-dried stem barks (200 g) of *T. catigua* were powdered and mixed with 1000 mL of distilled water. The extract was obtained by turbo-extraction (Ultra-turrax^®^ model UTC115KT, IKA, Campinas, Brazil), and the crude extract was filtered and stored at −20 °C in the dark. After, 1 mL of the plant extract was added into 10 mM AgNO_3_ solution (1000 mL) and incubated at 30 °C for 72 h in the dark. Morphology and size of bioAgNPs were determined by JEOL JEM 1400 transmission electron microscopy (JEOL, Tokyo, Japan) and dynamic light scattering using the Litesizer DLS 500 (Anton Paar, Graz, Austria). The structural analyses were performed by XRD on a PANalytical X’Pert PRO MPD diffractometer (Malvern Panalytical, Worcestershire, UK) [71]. The bioAgNPs were kept in the dark 4 °C to prevent aggregation and were stable for several months. The characteristics of the bioAgNPs are shown in Supplementary Figure S1.

For all antibacterial assays, EUG was dissolved in 8.0% DMSO to obtain a 42.72 mg/mL stock solution. A stock solution of 1.08 mg/mL bioAgNPs was prepared in ultrapure sterilized water. The stock solutions were maintained at −20 °C and were further diluted in the culture medium to obtain the concentrations used in each assay. DMSO did not exceed 1.0% in all assays. Medium plus 1.0% DMSO, and medium plus 1.0% DMSO plus bacterial cells were used as sterility and growth controls, respectively, in all assays.

### 3.2. Bacteria and Culture Conditions

*S. pseudintermedius* (*n* = 6) isolated from dogs diagnosed with keratoconjunctivitis sicca at the Veterinary Hospital of the Universidade do Oeste Paulista (UNOESTE, Presidente Prudente, Brazil) were included in this study. The study protocol was approved by the Ethics Committee for the use of animals of UNOESTE and was registered with the Research, Development, and Extension Coordination Office under number 3050/2016. The bacteria were stored in TSB containing 20% glycerol at −80 °C and kept in the bacterial collection of the Laboratório de Biologia Molecular de Microrganismos of the Universidade Estadual de Londrina, Londrina, Brazil. Bacterial isolates were inoculated onto MSA and incubated at 37 °C for 24 h. Suggestive colonies of staphylococci were subjected to phenotypic identification based on standard tests, including Gram staining, tube coagulase, catalase, urease, and carbohydrate fermentation assays [35]. The bacterial species was confirmed by multiplex PCR targeting the *nuc* gene (encoding thermonuclease) as described by Sasaki et al. [36]. The bacterial isolates were cultured in TSA at 37 °C for 24 h. The standard bacterial suspensions were prepared by transferring three to five colonies of each isolate into 0.15 M NaCl solution (saline) until achieving turbidity equivalent to a 0.5 McFarland standard (1.0–2.0 × 10^8^ CFU/mL) using the DensiCHECK™ PLUS colorimeter (bioMérieux, Rio de Janeiro, Brazil). The standard bacterial suspensions were further diluted in culture medium to achieve the inoculum used in each assay.

### 3.3. Antibacterial Activity Against Planktonic Cells

#### 3.3.1. Disk Diffusion

The antibacterial susceptibility profiles of the isolates were performed and interpreted using the disk diffusion assay as recommended by the Clinical and Laboratory Standards Institute guidelines [39]. *Staphylococcus aureus* ATCC 25923 was used as quality control. Oxacillin disk and/or the presence of *mecA* gene, detected as described by Milheiriço et al. [37], were used to define methicillin resistance [39].

#### 3.3.2. Minimum Inhibitory Concentration (MIC)

The MICs of EUG and bioAgNPs were determined by broth microdilution assay in U-bottom 96-well microtiter plates (Techno Plastic Products, Trasadingen, Switzerland) as recommended by the Clinical and Laboratory Standards Institute [39]. The stock solutions of EUG and bioAgNPs were two-fold serially diluted in CaMHB to achieve concentrations ranging from 0.02 to 10.68 mg/mL and 0.22 to 1024 μg/mL in the assay, respectively. MIC was defined as the lowest concentration capable of inhibiting visual bacterial growth after 24 h of incubation at 37 °C compared with growth control. The MBC for both compounds were determined by the time–kill assay, as described in Section 3.3.4. The inhibitory effect was interpreted according to the MBC and MIC ratio as follows: bactericidal MBC/MIC = 1–4; bacteriostatic MBC/MIC > 4 [41].

#### 3.3.3. Checkerboard Microdilution Assay

The antibacterial activity of EUG combined with bioAgNPs against planktonic cells was evaluated using the checkerboard broth microdilution assay, as described in Otaguiri et al. [30], with minor modifications. Two-fold serial dilutions of EUG (0.02–10.68 mg/mL) and bioAgNPs (0.22–1024 µg/mL) in CaMHB were, respectively, added across the rows and columns of the U-bottom 96-well microtiter plates. Around 1.0 × 10^5^ CFU/mL of bacterial cells were inoculated, and the plates were incubated at 37 °C for 24 h. The ratio between the MIC values of the compounds tested in combination and tested individually was used to calculate the fractional inhibitory concentration (FIC) of each compound. The sum of FIC_EUG_ and FIC_bioAgNPs_ was utilized to calculate the FIC index (FICI), whose values were interpreted as follows: synergistic FICI ≤ 0.5, no interaction 0.5 < FICI ≤ 4.0, or antagonistic FICI > 4.0 [55].

#### 3.3.4. Time–Kill Assay

The MBC and the rate of bacterial killing in the presence of EUG (0.67–5.34 mg/mL) and bioAgNPs (1.72–13.82 µg/mL) alone or in combination were evaluated by the time–kill assay [40]. Planktonic cells (5.0 × 10^5^ CFU/mL) were added to 2 mL of CaMHB containing EUG alone or bioAgNPs alone or EUG/bioAgNPs combination values. Bacterial growth in absence of the compounds was used as control. The cultures were incubated at 37 °C, and at specified time points (0, 2, 4, 8, 16, and 24 h), 20 µL was removed, ten-fold serially diluted, and each dilution was inoculated onto MHA. After incubation at 37 °C for 24 h, the CFU counts were carried out. Averaged data were plotted as log10 CFU/mL versus time (h). The bactericidal effect of the compounds was defined as a 99.9% (3 log10) reduction in CFU/mL of the starting inoculum [72].

#### 3.3.5. Cell Membrane Integrity

The effect of EUG alone or bioAgNPs alone on bacterial membrane integrity was evaluated using the LIVE/DEAD^®^ BacLight™ kit according to the manufacturer’s recommendations. Planktonic cells (1.5 × 10^7^ CFU/mL) were inoculated into 2.0 mL CaMHB containing EUG or bioAgNPs alone and the cultures were incubated at 37 °C for 2 h. Untreated and treated bacteria were incubated with propidium iodide (30 μM) and SYTO™ 9 (6 μM) at room temperature, for 15 min, and observed under a fluorescence microscope (OLYMPUS BX53, Tokyo, Japan) using a fluorescein filter with excitation/emission wavelengths of 480/530 nm, respectively. SYTO™ 9, a green fluorescent nucleic acid dye, stains live and dead bacteria, while propidium iodide, a red fluorescent nucleic acid dye, selectively stains bacteria with permeable (damaged) membranes.

### 3.4. Antibacterial Effect on Sessile (Biofilms) Cells

#### 3.4.1. Biofilm Production

Biofilm production capacity of all *S. pseudintermedius* isolates was evaluated in flat-bottomed 96-well polystyrene microtiter plates (Techno Plastic Products, Trasadingen, Switzerland) as described by Stepanovic et al. [60] with minor modifications. Briefly, 1.0 × 10^7^ CFU/mL (20 µL) was placed in each well containing 180 µL of TSB supplemented with 1% glucose, and the plates were incubated statically at 37 °C for 24 h. Afterward, non-adherent cells were removed by washing thoroughly two times with saline. Biofilm biomass was fixed with methanol (200 µL) for 15 min, dried at room temperature, and stained with 2% (*v*/*v*) crystal violet for 5 min. The biofilm-adhered stain was removed by the addition of acetic acid (160 µL) and transferred to another plate. The optical density (OD) was measured at 570 nm using a BioTek Synergy™ HT microtiter plate reader (Agilent, Santa Clara, CA, USA). The mean OD values of the negative controls plus 3 × standard deviations of the negative controls were used to define the cut-off OD (ODc). The isolates were classified as follows: ODtest ≤ ODc, non-producer; ODc < ODtest ≤ 2 × ODc, weak producer; 2 × ODc < ODtest ≤ 4 × ODc, moderate producer; 4 × ODc < ODtest, strong producer [60].

#### 3.4.2. Antibiofilm Activity

To determine the antibiofilm activity of the compounds, bacterial biofilms were formed as above. After incubation, the biofilms were washed twice with saline, and fresh medium (100 µL) containing different concentrations of EUG (0.33–5.34 mg/mL) or bioAgNPs (1.72–55.28 µg/mL) was added, and the biofilms were incubated at 37 °C for further 24 h. The biofilms were washed once with saline, and the biofilm biomass was determined as above.

As previous study showed that bioAgNPs do not interfere with the spectrophotometric analysis of the MTT reduction assay [32], this approach was utilized to analyze the effect of compounds on the metabolic activity of sessile cells. Therefore, after incubation, biofilms were washed with saline, and 100 µL of MTT (0.5 mg/mL)/menadione (0.5%) solution was added to each well, and the plates were incubated in the dark at 37 °C for 2 h. Formazan crystals were solubilized with a solution (100 µL) containing 10% Triton X-100, 0.1 N HCl in isopropanol, and then the OD was determined at 550 nm in a BioTek Synergy™ HT microtiter plate reader (Agilent, Santa Clara, USA). The MIC was determined as the lowest concentration capable of inhibiting 90% (SMIC_90_) of the metabolic activity of sessile cells compared to the untreated controls.

To evaluate the effect of the combination of EUG and bioAgNPs on the 24 h biofilm, it was formed as described above. After removing the non-adherent cells, two-fold serial dilutions of EUG (0.02–10.68 mg/mL) and bioAgNPs (0.22–1024 µg/mL) were added in the rows and in the columns, respectively, of the 96-well microtiter plates. The SMIC_90_ of the combination was determined by evaluating the metabolic activity of the sessile cells using the MTT reduction assay, as previously described. The FICI was calculated and the values interpreted according to the criteria established for planktonic cells.

Additionally, the viability of sessile cells of *S. pseudintermedius* SIG 3X and SIG 12X was determined by the CFU counts. Thus, untreated and treated biofilms were removed by scraping with a sterile scalpel. Sessile cells were resuspended in saline, vigorously vortexed for 30 s. An aliquot (20 µL) of the cell suspension was removed, ten-fold serially diluted, and 100 µL of each dilution was inoculated onto MHA. After incubation at 37 °C for 24 h, the CFU counts were carried out to estimate the total number of viable cells.

#### 3.4.3. Scanning Electron Microscopy (SEM)

The effect of compounds on morphology of biofilms was analyzed by SEM. Glass coverslip was immersed in wells of 24-well cell culture plates containing 1 mL TSB plus 1% glucose inoculated with *S. pseudintermedius* SIG 3X (1 × 10^6^ CFU), and the system was incubated at 37 °C for 24 h. The biofilms were washed twice with PBS and treated with EUG alone or bioAgNPs alone for 24 h at 37 °C. The biofilms were fixed with 2.5% (*v*/*v*) glutaraldehyde in 0.1 M sodium cacodylate buffer, pH 7.2 at room temperature for 4 h, dehydrated with serial ethanol washes (30%, 50%, 70%, 80%, 90%, and 100%), critical point dried using hexamethyldisilazane (HMDS) (BAL-TEC, CPD 030), coated with gold, and observed in a FEI Quanta 250 scanning electron microscope (Themo Fisher Scientific, Hillsboro, TX, USA).

### 3.5. Effect of Eugenol and Biogenic Silver Nanoparticles on Mammalian Cells

The cytotoxicity of EUG and bioAgNPs was evaluated on kidney epithelial cells from *Macaca mulatta* [LLC-MK2 cells (Merck, Brazil)]. Cells (5 × 10^4^ cells/well) were cultured in DMEM with 10% fetal bovine serum, 2 mM L-glutamine, 100 IU/mL penicillin, 100 µg/mL streptomycin, 1% tylosin in flat-bottomed 96-well microtiter plates, in 5% CO_2_ at 37 °C for 48 h. The medium was aspirated off and fresh medium containing EUG (0.08–5.34 mg/mL) or bioAgNPs (0.10–13.82 µg/mL) was added. The cells were incubated for further 24 h under the same conditions. Cell viability was analyzed by the MTT reduction assay according to the manufacturer’s recommendation. The concentration of the compound needed to inhibit the viability of 90% of the cells calculated by regression analysis corresponds to the 90% (CC_90_) cytotoxic concentrations.

### 3.6. Statistical Analyses

All the experiments were carried out in at least two biological replicates at least three different occasions, and the results are expressed as the mean ± standard deviation. The GraphPad PRISM software version 8.0 (GraphPad Software, San Diego, CA, USA) was used for statistical analyses. Time–kill kinetics and toxicity to LLC-MK2 cells were analyzed using two-way ANOVA followed by Tukey’s multiple comparisons test. The biofilm results were evaluated by one-way ANOVA. For all assays, *p* < 0.05 was considered significant.

## 4. Conclusions

This study investigated the antibacterial effect of eugenol (EUG) and biogenic silver nanoparticles (bioAgNPs) synthesized using an aqueous extract of *Trichilia catigua* A. Juss. bark, both alone and in combination, against planktonic and sessile cells of multidrug-resistant *Staphylococcus pseudintermedius* isolated from canine keratoconjunctivitis sicca. The main findings include the following: (i) EUG and bioAgNPs showed dose- and time-dependent bactericidal activity against planktonic cells, thereby interfering with cell membrane integrity; (ii) EUG and bioAgNPs showed a synergistic antibacterial effect towards planktonic cells of most isolates tested—although the nature of this interaction was classified as bacteriostatic, it resulted in a four-fold reduction in the MIC values of both compounds; (iii) EUG and bioAgNPs led to a significant dose-dependent reduction in the metabolic activity and biomass of 24 h biofilms formed on the surface of polystyrene and glass, promoting severe morphological changes in sessile cells; (iv) the EUG and bioAgNPs combination also inhibited pre-formed (24 h) biofilms; (v) EUG and bioAgNPs exhibited reduced toxicity toward LLC-MK2 cells at concentrations corresponding to synergistic or indifferent combinations.

This study has some limitations that may restrict the generalizability of the results. The small number of clinical isolates evaluated does not cover the full diversity of genetic and antimicrobial susceptibility profiles of *S. pseudintermedius*. Furthermore, most of the experiments were conducted under in vitro conditions, which may not accurately reflect the complexity of infectious environments in vivo. Another point to consider is that the mechanism of action of the EUG and bioAgNP combination has not yet been fully elucidated. Despite these limitations, our findings indicate that the combination between EUG and bioAgNPs constitutes a promising and safe strategy, with the potential to be used as an alternative or adjuvant therapy in the control of infections caused by multidrug-resistant strains of *S. pseudintermedius*. In addition, the use of green routes for the synthesis of nanoparticles contributes to the sustainability of the process by avoiding the use of toxic reagents, which aligns with the principles of green chemistry and current demands for environmentally responsible technologies.

## Figures and Tables

**Table 1:** Characterization of *Staphylococcus pseudintermedius*: antimicrobial resistance profile and antibacterial activity of eugenol (EUG) and biogenic silver nanoparticles (bioAgNPs).

Isolate	Resistance profile^a^	*mec*A^b^	EUG		BioAgNPs		MBC/MIC^e^	
			MIC^c^	MBC^d^	MIC^c^	MBC^d^	EUG	bioAgNP
SIG 3X	OX, P, AZM, ERY, DA*, CIP, LEV, CN*, TE, CHL	positive	1.33	2.67	3.45	13.82	2.0	4.0
SIG 4X	OX, P, AZM, ERY, DA*, CIP, LEV, CN, CHL	positive	0.67	1.33	3.45	13.82	2.0	4.0
SIG 9X	OX, P, AZM, ERY, DA*, CIP, LEV, CN*, TE, CHL	positive	1.33	1.33	3.45	13.82	1.0	4.0
SIG 10X	OX, P, AZM, ERY, DA*, CIP, LEV, CN*, TE, CHL	positive	1.33	1.33	3.45	6.91	1.0	2.0
SIG 11X	P, AZM, ERY, DA*, CIP, LEV, CN*, TE	negative	0.67	1.33	3.45	6.91	2.0	2.0
SIG 12X	P, AZM, ERY, DA*, CIP, LEV, TE, CHL	negative	1.33	2.67	3.45	6.91	2.0	2.0

^a^Phenotypic resistance was determined by the disk diffusion method [39]. AZM: azithromycin; CHL: chloramphenicol; CIP: ciprofloxacin; DA: clindamycin; ERY: erythromycin; CN: gentamicin; LEV: levofloxacin; OX: oxacillin; P: penicillin; TE: tetracycline. ^b^The presence of the *mecA* gene was determined through PCR [37]. ^c^The minimum inhibitory concentration (MIC) was determined by the broth microdilution method [39]. ^d^The minimum bactericidal concentration (MBC) was determined by the time–kill assay [40]. ^e^Reference values: 1 to 4 bactericidal effect and > 4 bacteriostatic effect [41]. *Intermediate resistance. EUG values are expressed in mg/mL; bioAgNPs values are expressed in µg/mL.

**Table 2:** Antibacterial activity of eugenol (EUG) combined with biogenic silver nanoparticles (bioAgNPs) against planktonic cells of MDR *Staphylococcus pseudintermedius*.

	MIC				
Isolate	EUG^a^	bioAgNPs^a^	EUG/bioAgNPs^b^	FICI^c^	Interaction^d^
SIG 3X	1.33	3.45	0.33/0.86	0.50	Synergism
SIG 4X	0.67	3.45	0.16/0.86	0.49	Synergism
SIG 9X	1.33	3.45	0.33/1.72	0.75	Indifferent
SIG 10X	1.33	3.45	0.33/1.72	0.75	Indifferent
SIG 11X	0.67	3.45	0.16/0.86	0.49	Synergism
SIG 12X	1.33	3.45	0.33/0.86	0.50	Synergism

^a^The minimum inhibitory concentration (MIC) determined by the broth microdilution method [39]. ^b^The MIC of EUG and bioAgNPs in combination determined by checkerboard assay [30]. ^c^The fractional inhibitory concentration index (FICI) was calculated as the sum of the FIC_EUG_ and FIC_bioAgNPs_. ^d^Reference values: synergism (FICI ≤ 0.5), no interaction (0.5 < FICI ≤ 4.0), or antagonism (FICI > 4.0) [55]. EUG values are expressed in mg/mL; bioAgNPs values are expressed in µg/mL.

**Table 3:** Antibiofilm activity of eugenol (EUG) and biogenic silver nanoparticles (bioAgNPs), alone and in combination, against sessile MDR *Staphylococcus pseudintermedius*.

	SMIC_90_				
Isolate	EUG^a^	bioAgNPs^a^	EUG/bioAgNPs^b^	FICI^c^	Interaction^d^
SIG 3X	1.33	13.82	0.33/3.45	0.50	Synergism
SIG 4X	1.33	13.82	0.33/3.45	0.50	Synergism
SIG 9X	1.33	13.82	0.33/3.45	0.50	Synergism
SIG 10X	0.67	13.82	0.33/3.45	0.74	Indifferent
SIG 11X	0.67	13.82	0.16/3.45	0.49	Synergism
SIG 12X	1.33	13.82	0.33/3.45	0.50	Synergism

^a^SMIC_90_: Sessile minimum inhibitory concentration capable of inhibiting 90% of the metabolic activity of sessile cells. ^b^SMIC_90_ of EUG and bioAgNPs in combination determined by checkerboard assay [30]. ^c^FICI: fractional inhibitory concentration index was calculated as the sum of the FIC_EUG_ and FIC_bioAgNPs_. ^d^Reference values: synergism (FICI ≤ 0.5), no interaction (0.5 < FICI ≤ 4.0), or antagonism (FICI > 4.0) [55]. EUG values are expressed in mg/mL; bioAgNPs values are expressed in µg/mL.

## Data Availability

The original contributions presented in this study are included in the article/Supplementary Material. Further inquiries can be directed to the corresponding author.

## Supplementary Materials

The following supporting information can be downloaded at: https://www.mdpi.com/article/10.3390/molecules30163353/s1, Figure S1: Characteristics of biogenic silver nanoparticles synthesized (bioAgNPs) using the aqueous extract of *Trichilia catigua* A. Juss. bark. Figure S2: Effect of eugenol (EUG) and biogenic silver nanoparticles (bioAgNPs) on the cell membrane integrity of multidrug-resistant *Staphylococcus pseudintermedius* planktonic cells; Figure S3: Biofilm biomass of multidrug-resistant *Staphylococcus pseudintermedius* formed on polystyrene surface after 24 h of incubation at 37 °C.

## Abbreviations

The following abbreviations are used in this manuscript:

MDR	Multidrug resistant
KCS	Keratoconjunctivitis sicca
MRSP	Methicillin-resistant *Staphylococcus pseudintermedius*
EUG	Eugenol
AgNPs	Silver nanoparticles
bioAgNPs	Biogenic silver nanoparticles
PCR	Polymerase chain reaction
MSSP	Methicillin-sensitive *Staphylococcus pseudintermedius*
MIC	Minimum inhibitory concentration
MBC	Minimum bactericidal concentration
CFU	Colony forming unit
ROS	Reactive oxygen species
FICI	Fractional inhibitory concentration index
OD	Optical density
SMIC	Sessile minimum inhibitory concentration
MTT	3-(4,5-dimethylthiazol-2-yl)-2,5-diphenyltetrazolium bromide
DMSO	Dimethyl sulfoxide
MSA	Manitol Salt Agar
TSB	Tryptone Soya Broth
CaMHB	Cation-Adjusted Müller Hinton Broth
MHA	Müller Hinton Agar
SEM	Scanning electron microscopy
DMEM	Dulbecco’s Modified Eagle medium
