# Supplementary material for: Synergistic Antibacterial Effect of Eugenol and Biogenic Silver Nanoparticles on Staphylococcus pseudintermedius Isolated from Canine Keratoconjunctivitis Sicca
Source: Molecules. 2025 Aug 12;30(16):3353. doi: 10.3390/molecules30163353 (PMC12388120; doi:10.3390/molecules30163353)
Supplement: Supplementary file 1 [file molecules-30-03353-s001.zip › molecules-3776385-supplementary--updated.pdf]

molecules

MDPI

Article

Synergistic Antibacterial Effect of Eugenol and Biogenic Silver Nanoparticles on *Staphylococcus pseudintermedius* Isolated from Canine Keratoconjunctivitis Sicca

Weslei Roberto Correia Cabral[1], Caio Ferreira de Oliveira[2], Rogerio Giuffrida[3], Lais Fernanda de Almeida Spoladori[1], Guilherme Bartolomeu-Gonçalves[4], Helena Tiemi Suzukawa[5], Gabriella Maria Andriani[1], Gerson Nakazato[1], Eliandro Reis Tavares[1,5,6], Lucy Megumi Yamauchi[1,5] and Sueli Fumie Yamada-Ogatta[1,4,5,*]

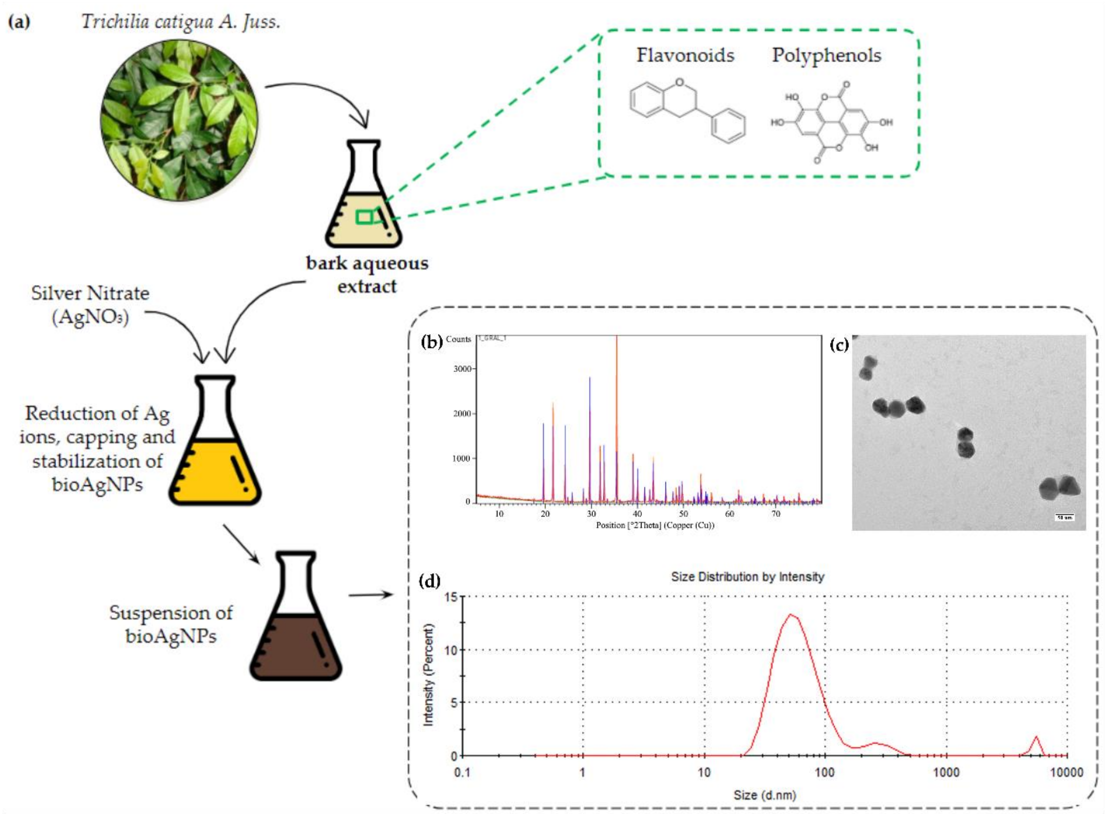

Figure S1: Characteristics of biogenic silver nanoparticles (bioAgNPs) synthesized using the aqueous extract of Trichilia catigua Adr. Juss bark. (a) Biogenic silver nanoparticles (bioAgNP) were obtained after AgNO3 reduction by the aqueous extract of T. catigua A. Juss bark, a process that was patented (BR 102021016375-5) [25]. The bark was acquired from GRAL Bioativos® LDTA (Nano VerdeAg®, Londrina, Brazil). (b) Structural analyses were performed by XRD on a PANalytical X'Pert PRO MPD diffractometer (Malvern Panalytical, Worcestershire, The United Kingdom) [71]. X-ray diffraction (XRD) analyses of bioAgNPs were carried out at the Laboratory of X-ray Analysis (LARX) of the Universidade Estadual de Londrina. The data was obtained on a PANalytical diffractometer, model X'Pert PRO MPD (Malvern Panalytical, Worcestershire, The United Kingdom) operating with CuKα radiation (λ = 1.5406 Å), using the θ-2θ geometry. The equipment's operating conditions were 40 kV voltage and 30 mA current. The samples were analyzed in the 2θ range between 5° and 80°, with an angular step of 0.04° and a counting time of 6.0 s per point. In order to minimize preferential orientation effects and ensure greater representativeness of the analyzed surface, the samples were subjected to continuous rotation during data acquisition, with a period of 1 s per cycle. (c) Morphology and dispersion determined by JEOL JEM 1400 transmission electron microscopy (JEOL, Tokyo, Japan) [71]. (d) Size of bioAgNPs was determined by dynamic light scattering using the Litesizer DLS 500 (Anton Paar, Graz, Austria) [71].

molecules

MDPI

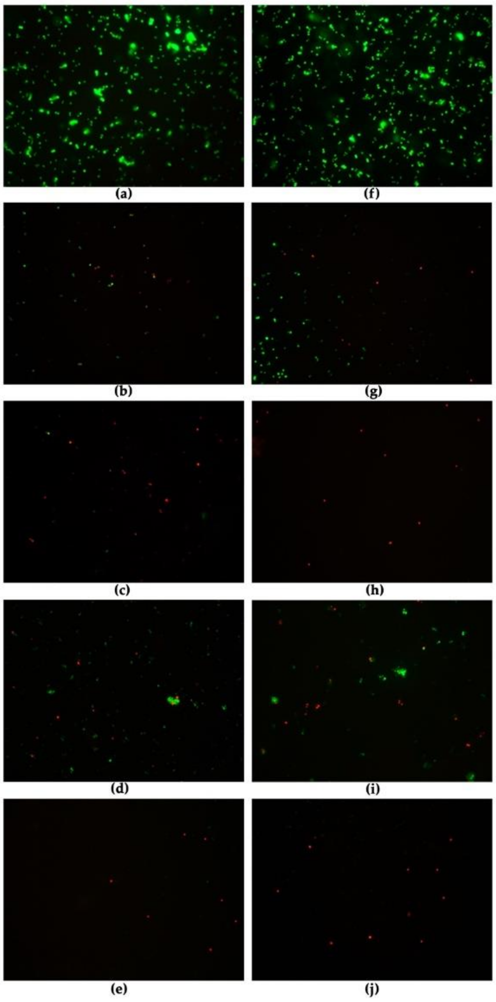

Figure S2: Effect of eugenol (EUG) and biogenic silver nanoparticles (bioAgNPs) on the cell membrane integrity of multidrug-resistant Staphylococcus pseudintermedius planktonic cells. Bacterial cells were incubated in absence of compounds (a,f), with EUG MIC (b,g) and MBC (c,h), and bioAgNPs MIC (d,i) and MBC (e,j) for 1 h. Viability analysis of planktonic cells after differential labeling with SYTO9™ and propidium iodide. The green fluorescence represents metabolically active bacteria, and red fluorescence indicates metabolically inactive bacteria with damaged membranes. (a – e) S. pseudintermedius SIG 3X; (f – j) S. pseudintermedius SIG 12X. 1000 × magnification.

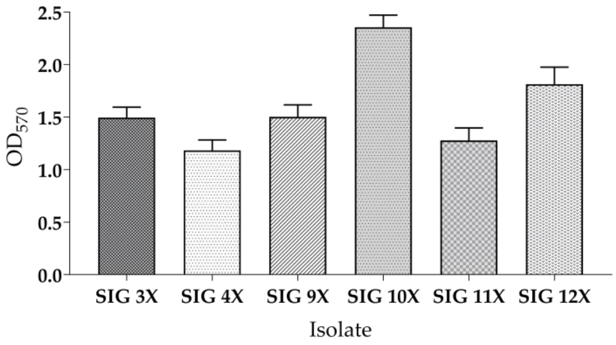

(a)

Isolate	OD ± sd	Category
SIG 3X	1.50 ± 0.10	Strong
SIG 4X	1.18 ± 0.10	Strong
SIG 9X	1.50 ± 0.11	Strong
SIG 10X	2.36 ± 0.12	Strong
SIG 11X	1.28 ± 0.12	Strong
SIG 12X	1.81 ± 0.16	Strong

(b)

Figure S3: Biofilm biomass of multidrug-resistant Staphylococcus pseudintermedius formed on polystyrene surface after 24 h of incubation at 37 °C. The biofilms were formed on flat-bottomed 96-well microtiter plates in TSB supplemented with 1% glucose, and the biomass (a) was quantified using a 2.0% crystal violet-based

assay [60]. Thus, after crystal violet staining, the biofilms were washed twice with sterilized saline, and the biofilm-bound stain was removed by the addition of 95% methanol. The bleaching solution was transferred to another plate, and the optical density (OD) was measured at 570 nm with a microtiter plate reader BioTek Synergy™ HT microtiter plate reader (Agilent, Santa Clara, USA). (b) The mean OD values were interpreted according to the criteria proposed by Stepanovic et al. [60].
